# To Comply or Not to Comply: Roma Approach to Health Laws

**DOI:** 10.3390/ijerph17093087

**Published:** 2020-04-29

**Authors:** Barbara Pavlikova, Lenka Freel, Jitse P. van Dijk

**Affiliations:** 1 Research Agency, 831 02 Bratislava, Slovakia; 2 Department of Labor Law and Social Welfare Law, Faculty of Law, Comenius University, 810 00 Bratislava, Slovakia; 3 Department of Community and Occupational Medicine, University Medical Center Groningen, University of Groningen, 9713 AV Groningen, The Netherlands; 4 Graduate School Kosice Institute for Society and Health, Faculty of Medicine, P.J. Safarik University in Kosice, 040 01 Kosice, Slovakia; 5 Theological Faculty, Olomouc University Social Health Institute, Palacky University, 771 11 Olomouc, Czech Republic

**Keywords:** Roma health, Slovakia, non-compliance

## Abstract

According to the general public in Slovakia, compliance with the law is problematic when it comes to Roma and health. Roma compliance with laws has not yet been studied. The aim of this is study was to explore the determinants of Roma behavior in the field of health laws. We used the concept of a semi-autonomous field proposed by Moore (1973) and the theory of planned behavior by Ajzen (1985). We found that Roma (non-)compliance with health laws was influenced by many different factors, such as beliefs, traditions, living conditions and culture. Group beliefs overrule national laws and also individual preferences, which tend to be subordinate to the group view. The less contact Roma from settlements have with non-Roma, the stronger their own rules are in the field of health. Roma health status is influenced by many factors: group beliefs and community traditions are stronger and overrule individual and state behavioral influence. A community-based participatory approach together with improvement of living conditions in cooperation with Roma is desirable.

## 1. Introduction

### 1.1. Roma Health

The Roma population is the largest ethnic group in Europe [1] and is still widely exposed to poverty, discriminatory approaches and social exclusion, while facing an unprecedented level of discrimination in comparison with any other ethnic groups living in the same territory [2,3,4]. One in four Roma said in 2016 that they had experienced discrimination within the last year, but only about 10 percent reported it to authorities [5]. The lifestyle of the Roma minority in Slovakia differs from the non-Roma majority and is the result of both internal and external factors that are closely connected [6]. Roma experience severe health inequalities and are considered to be a “hard to reach“ group [7]. They also suffer from a high risk of poor health and low levels of well-being in comparison with the majority population [8,9,10]. Some of the reasons lie in an unhealthy lifestyle, featuring heavy smoking, poor nutrition and housing [11,12,13,14]; others lie in the Roma approach to a range of health issues, but social exclusion, discrimination and segregation caused by the majority were all mentioned as the main external factors having a strong influence on the health status of Roma [15,16]. Far-right political populism in Europe widely supports and strenghtens negative external factors [17].

Roma often encounter various barriers when accessing healthcare services, resulting in negative health outcomes [18,19,20]. Ethno-cultural and socioeconomic factors also have a negative influence on the health status of Roma [21]. Cultural and structural factors influence the engagement of marginalised populations in regard to health services and preventive programs [22,23]. Societal infrastructure is another important determinant of their health status [24,25]. An intercultural approach seems to be necessary to address the needs of this ethnic group [26]; however, systematic studies of Roma health are lacking [27].

### 1.2. Semi-Autonomous Fields

Moore [28] explains the concept of a semi-autonomous field as an appropriate subject of study, because small selected fields generating rules, customs and symbols internally, but still vulnerable to rules and decisions from the external environment, have rule-making capacity and are able to induce or coerce compliance. Those minority social groups that exist in every complex society have something that Weber [29] calls a “legal order” and are able to create their own rules. Group rules can sometimes appear to be superior to the national or state rules and can also override them, so the group acts as if no government exists. The semi-autonomous field itself represents a form of “governance” [30]. Autonomy and isolation are the core concepts of this theory [28]. The Roma minority in Slovakia represents such a semi-autonomous field.

### 1.3. Compliance

In recent years, adherence to medical recommendations has already become the subject of expert studies [9,31]. The enhancement of access to health services and trustful relationship with leaders of the community is essential to ensure adherence [32]. However, the concept of compliance with the law in relation to health behavior and interventions in the area of public health is a new approach, and evidence on this is totally lacking. 

The concepts of “compliance” and “non-compliance” come from international law [33,34,35,36,37], while healthcare is one of the main concerns of social sciences [38]. Trainor [39] understands compliance as an individual’s or a group’s conformity with or obedience to a set of rules or regulations determined by the law or any governing body. It can include group (or internal) rules and state laws [40]. When a new regulation in some specific area appears, it may be a response to criminal or negligent behavior, or it can serve as extra protection [41]. In general, compliance means the willingness to act according to an order, a set of rules or a request [42]. Several opinions on compliance are circulating [43,44]; in this case, we adhere to Von Stein [45]. She understands compliance as the degree to which state (national or lower) behavior conforms to what the agreement prescribes or proscribes. Non-compliance will then be defined as conflict with such a prescription (expected behavior).

In our previous work [46], we discussed compliance with the law from the state point of view. We examined Slovakia with regard to respect for international commitments in building a smoking-free environment. We found that Slovakia (compared to Finland) has a problem with the absence of effective structural support, funding and realistic identification of gaps in the implementation of international commitments. These were the most problematic areas and the biggest barriers to achieving better results. 

Information is rather lacking on the concept of compliance with the law at the level of an ethnic group, regarding its health behavior and interventions in the field of public health. Therefore, the aim of this study was to explore the determinants of Roma behavior in the field of legal norms for health. 

## 2. Materials and Methods 

### 2.1. Sample

Our design was an analytical study, with the studies of Ajzen (The Theory of Planned Behavior) and of Moore (Semi-autonomous field) as the most important starting points. We worked predominantly with publications explaining the concept of semi-autonomous fields (SAF) [28] and the theory of planned behavior (TPB) [47] and with Belak’s papers on Roma [25,48,49]. Documents were obtained mainly from scientific articles published in reputable journals, as listed in the references. The dissertation of researcher Andrej Belak was a special source, because it provides a wide range of information based on long-lasting experience from Roma settlements. The Second European Union Minorities and Discrimination Survey was a source for information together with the work of the Poverty and Equity Global Practice and the Social Protection and Jobs Global Practice (the World Bank). Slovak and EU statistics are not cited directly in the manuscript, as we need them to acquire a better idea of the status of Roma all over Europe. Official websites of political authorities and laws in force were also used to check the information. These can be found in the official Collection of Laws [50] and in the official database of EU legislation [51]. No Ethics Committee approval was necessary for our documentary study.

Roma are one of the largest and internally most variable ethnically defined populations in Europe [48]. Due to this, it is very difficult to identify some general characteristics. We focused mainly on Roma in segregated settlements in Slovakia, because in the context of healthcare, they are the most interesting group to focus on, and they also face the widest discrimination in many areas.

### 2.2. Measures

**Figure 1:**
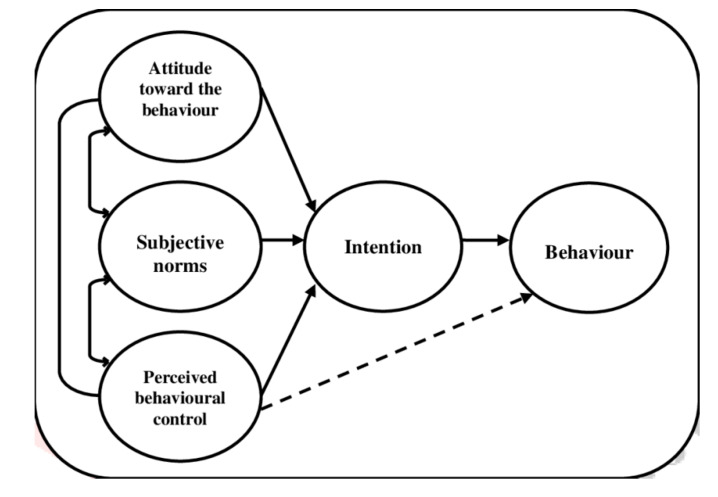
Theory of planned behavior [52].

We used the concept of a semi-autonomous field to explain the relationship between state and group norms [28]. We also used the TPB developed by Ajzen [47], which represents an extension of the theory of reasoned action (Figure 1). 

We measured the concept of (non-)compliance with the law through the lens of the concept of intention to comply. Its precursors, namely attitude, subjective norm and perceived behavioral control, were discussed. We measured attitude through cognition, values and affection; subjective norm through perceived social pressure and perceived behavioral control through beliefs about resources and opportunities.

This direction was also crucial while searching for the scientific sources and information needed for adopting conclusions based on field research. We concentrated particularly on publications that provide us enough information appropriate for their assessment in the light of mentioned concepts of SAF and TPB. 

### 2.3. Reporting

The aim of this study was to explore the determinants of Roma behavior in the field of legal norms for health by using the concepts of a semi-autonomous field by Moore and the Theory of Planned Behavior by Ajzen. We considered this to be an innovative way of looking at factors influencing the way Roma behave when it comes to compliance with health laws. We focused on internal and external factors that influence the health status of Roma and their attitudes regarding compliance issues. We discussed them in the light of the mentioned theories and tried to pay attention to some new point of view which may make it easier for policy-makers to take into account the strength and weight of “behind the scene” determinants when discussing political, economic and medical measures for improving the status of Roma.

We first focused on the description of the Roma minority as a semi-autonomous field [28] in the context of factors influencing its compliance with health laws. We continued with intention as one of the influencing factors according to Ajzen. We next paid attention to attitude toward behavior understood as a group and as a personal factor. An answer will be given to the question of whether it is the group’s (Moore) or the individual’s (Ajzen) positive or negative evaluation of performing the behavior. Then, we dealt with the concept of subjective norm as the group’s and as the person’s perception of the social pressures put on them to perform or not perform the behavior the (state or group) law requires, i.e., perceived behavioral control. People tend to engage in a behavior when they evaluate it positively and when they believe that important others think they should engage in it [47]. We will see whether this is especially true in the case of members of ethnically defined semi-autonomous groups. 

## 3. Results

### 3.1. Factors Influencing Roma (Non-)Compliance with Health Laws

The more remote a Roma settlement is from urban centers, the more closed its residents become, and they transform themselves into a semi-autonomous social group (SASG) [53]. A SASG can be defined among other things by its autonomy and isolation [28]. Both characteristics support and strengthen the tendency to self-governance and the establishment of group norms, which often overrule the state’s generally-binding legislation, including health laws. The Roma minority generates rules, customs and symbols internally; develops rule-making capacity and has the means to induce or coerce compliance with generated rules and customs in its own way, outside the official political system [28]. We can even say that each fajti (kinship) creates a single SASG, and members of one fajti are very competitive and hostile to others, which leads to their isolation from each other and from non-Roma [25]. 

There are a number of factors influencing the willingness and the ability of Roma to comply with health laws imposed by the state, such as compulsory vaccination, preventive examinations or legislation focusing on healthy lifestyle. Ethnically framed pro-non-compliance norms, Roma fatalism, their fear of being misunderstood by the majority and socioeconomic and other ethno-cultural factors can be included [25,54]. Additionally, strong anti-Roma prejudice remains a proven barrier to efforts to improve the living conditions and health status of Roma [55,56,57].

According to researchers [49], Roma themselves have adopted racialized arguments and ethnically framed social counter-norms, saying that they differ because of capacities embodied in their “blood”, “brains”, “bodies” or “genes” or that they have a specific esthetic style. However, these ethnically framed norms were not found to apply among children and the elderly [25]. 

Material and living conditions also play a significant role in compliance with health laws and are in most cases beyond the control of Roma themselves. The unhealthy environment and lack of personal hygiene resulting from poor housing conditions (mainly the lack of water mains, heating and sanitation) [58,59,60] aggravate the stigmatization and often contribute to ignoring the requirements defined in national legislation. Together with psychosocial factors leading to chronic stress, such as lack of money, the need to go out of the settlement, occasional physical violence and dependence on social welfare payments, this creates a closed circle. The consequences of such a circle are then reflected in higher smoking rates, higher alcohol consumption, an unhealthy diet or a prevailing unhealthy lifestyle in general, although the first two above-mentioned behaviors in particular were not found in Roma adolescents [61]. 

### 3.2. Intention—Is Roma Compliance in the Field of Health Goal-Oriented?

An intention consists of three conceptually independent determinants: attitude, subjective norm and perceived behavioral control. The more favorable the attitude and the subjective norm regarding a certain behavior, and the greater the perceived behavioral control and the stronger an individual’s intention to perform this behavior should be. The relative importance of attitude, subjective norm and perceived behavioral control in the prediction of the intention is expected to vary across behaviors and situations [62]. People’s intention indicates how strongly they are willing to perform that behavior, which should be under their volitional control. Final success depends on intention and ability (behavioral control). 

When applying this way of thinking to Roma compliance with health laws, we can assume that non-compliance can be caused either by a lack of intention to perform a certain behavior, e.g., refusing to undergo compulsory vaccination because of some fatalistic beliefs or by a misunderstanding of the purpose of the vaccination or by lack of ability to comply, e.g., due to a lack of resources to take the child to the healthcare facility, lack of personal hygiene or the impossibility of leaving other children alone at the settlement. 

Diversion from national health laws is caused by internal reasons in the first place, such as individual or group beliefs, or by living conditions that create contact barriers. Roma must have the same desire as non-Roma, i.e., they also want to be healthy, but their reasons and ways of achieving well-being often differ. However, we cannot ignore the ongoing discrimination and the support of the majority for strengthening the segregation and exclusion of Roma—especially those suffering from generational poverty—from public and societal life.

#### 3.2.1. Attitude—Group or State Laws?

Attitude refers to the degree to which a person has a favorable or unfavorable evaluation or appraisal of the behavior in question [62]. Attitude is a psychological construct which is shaped by cognition (thought), values (beliefs) and affection (emotions) toward a particular object [63]. In the case of attitudes towards a certain behavior, each belief links the behavior to a certain outcome or to some other attribute, such as the cost incurred by (not-)performing the behavior. Since the attributes which come to be linked to the behavior are already valued positively or negatively, people automatically and simultaneously acquire an attitude towards the behavior. They learn to favor behaviors they believe to have largely desirable consequences and form unfavorable attitudes towards behaviors which are associated with mostly undesirable consequences [62]. 

Taking into account all the factors mentioned in Section 3.1, we consider the psychosocial factors and ethnically framed social counter-norms, together with health behavior and health utilization, to be most influential in relation to attitude. Not only the need to leave the settlement to fulfil some medical requirements and the lack of funds to do so, but also, the approach of medical professionals affected by racist prejudices often cause long-lasting stress for Roma [58]. Preventive measures and post-recovery therapy have only a weak tradition in settlements [48], and this, together with unhealthy lifestyle, does not go hand-in-hand with compliance with national health laws. Healthcare facilities are usually far from the settlement, and visiting them not only costs time but also money. Poor accessibility and affordability of the healthcare services also do not support compliance with health laws, further promoting discrimination and exclusion [64]. To make Roma more compliant, it would be necessary to overcome not only the thoughts, beliefs and emotions of the individuals but also the group rules, which are in this case stronger than the affiliation to the state. 

#### 3.2.2. Subjective Norm—Social Pressure in a Semi-Autonomous Field

A subjective norm refers to the perceived social pressure to perform or not to perform a certain behavior [62]. Norms are determined by the perceived social pressure from others for an individual to behave in a certain manner and the individual’s resulting motivation to comply with those people’s views [65].

This field is influenced by the overruling group norms, beliefs and traditions in the widest range. Roma are not inclined to share their points of view, because they automatically assume that non-Roma will consider them as irrational and inexcusable [48]. Compliance with the rules in the educational sphere, long-term employment or healthcare, for example, are ridiculed and considered as being “too-non-Roma-like”. Roma who are not compliant are the “true ones”. Roma especially in segregated settlements have only limited contact with the non-Roma population, and due to their poor living conditions, they are to a large extent dependent on support from their kinship. This fact strengthens the group influence. The beliefs, traditions and perspective of group members prevail over state requirements [66]. One of the most important reasons is the fact that the Roma are an ethnic group facing systematic, targeted and, despite all declared effort, still unsolved discrimination [67] and segregation at every stage of their life, from the start of their education through efforts to get a job and to undergo a health examination. It is understandable that their trust into the state and national authorities is considerably weakened.

#### 3.2.3. Perceived Behavioral Control—When the Prior Experience of Roma Shapes Their Future Behavior

Perceived behavioral control refers to the perceived ease or difficulty of performing the behavior, and it is assumed to reflect past experience as well as anticipated possibilities and/or obstacles [62]. This concept usually varies across situations and actions, and it creates a framework of relations among beliefs, attitudes, intentions and behavior [68]. The more resources and opportunities individuals believe they possess, and the fewer obstacles or impediments they anticipate, the greater their perceived control over their behavior should be. As beliefs concerning the consequences of a certain behavior are viewed as determining attitudes toward that behavior, and normative beliefs are viewed as determining subjective norms, so beliefs about resources and opportunities are viewed as underlying the perceived behavioral control [62]. 

Roma themselves are affected by racist views claiming that poorer health and lower socioeconomic status reflect specific natural incapacities of the Roma. They have adopted racialized arguments [49]. This approach assumes fewer resources and opportunities and, as a consequence, a reduced willingness to engage in expected behaviors. A fatalistic view, their own belief in being less capable of performing a certain behavior due to their socioeconomic status together with health-access barriers create obstacles to compliance with health laws. 

## 4. Discussion

We explored the determinants of Roma behavior in the field of legal norms for health. We found that non-compliance with health laws can be caused by lack of intention or ability to engage in expected behaviors. Our findings show that the diversion from national health laws is caused by internal, as well as external, reasons beyond Roma control, whereby individual and group beliefs overrule state legal requirements. Psychosocial, socioeconomic factors and ethnically framed norms, together with racialized arguments adopted by Roma themselves caused by long-lasting discrimination, segregation and social exclusion by the majority and political representatives, seem to be the most important reasons of non-compliance with health laws.

We found that culture and traditions play a significant role in Roma lives. These different points of view often meet with institutional requirements that do not always respect them. Schneeweis [69] discusses the role of institutional influence in relation to cultural norms. She points out the importance of the cultural sensitivity framework and participatory paradigm. For instance, if more sensitive language and communication tools are used in communication with the targeted group, the health measures are more effective. A culture-centered approach to health communication strengthens the mutual relationships and supports better implementation of health laws. 

We further found that institutional domination is widespread. The top-down model is preferred by institutions in search of “good” Roma, which often even further reinforces prejudices on both sides [66]. The greater attention paid by the European Union towards the integration of Roma sometimes brings more institutionalization, which asserts domination over cooperation. Authorities such as doctors, mayors or social workers exert influence across physical borders and over Roma homes. Space is organized to keep the Roma in their place, secluded in their settlements, monitored and monitorable [69]. Resources organized by state intervention often lead to a physical and symbolic marginalization and ignore the cultural norms and real living conditions within settlements [70].

### 4.1. Factors Influencing Roma Compliance

We found that poor living and material conditions can be very influential with regard to compliance. The different and typically worse health statuses of Roma living in segregated settlements is related to the higher prevalence of unhealthy lifestyle [71,72]. Unhealthy lifestyle is closely associated with the living environment [73,74]. When ensuring compliance with health laws, it is also crucial to provide universal access to healthcare and universal health coverage, because accessibility of care is one of the factors identified as a potential barrier [26,75,76]. In Slovakia, equal coverage for everyone officially exists; therefore, ethnic differences must have another origin. All these factors influence intention and its elements in a positive or negative way. Children copy the behavior of their parents, and all members of the kinship or community together shape the motivation, beliefs, subjective norms and idea of desirable behavior for current and future generations. 

### 4.2. Intention

We found that when group rules promote behavior that is different from national laws, then motivation is obviously more in favor of the group rules. In addition to this primary condition, it can be added that if external factors, such as perceived barriers, further support the group adjustment, then the ability to comply with national health laws is very rare.

Roma are often described as helpless, needing intervention, lacking the skills and knowledge to improve their lives and without a future-oriented perspective or as uneducated, underprivileged and isolated simple-minded people with too many children [69]. This, together with external factors influencing Roma daily life, which they cannot influence on their own, creates an even greater distance between their intentions and intentions of the majority.

#### 4.2.1. Attitude 

We further found that Roma (non-)compliance is a persisting attitude among the privileged compared to the target population. If anti-Roma prejudice is still present, then members of marginalized communities as members of an SASG will constantly hit an invisible ceiling in their attempts to escape from their unfavorable situation and overcome the complex consequences of social exclusion [77]. Intergroup contact with Roma people is associated with more negative attitudes, despite all the official anti-discriminatory measures [56]. These attitudes have cultural roots in both the Roma and the majority. Any measures in the field of compliance with health laws should be able to merge healthcare and culture; otherwise, the results will be weaker [19,78]. 

Next, we found that an equal and cooperative approach is missing. A participatory paradigm, as presented by Schneeweis [69], paying attention to cultural traditions and allowing Roma to express their health choices, preferences and needs, may be a possibility for keeping traditions alive while seeking to implement changes desirable for Roma and their health status. This concept is also committed to changing behavior [69]; however, it focuses on cooperation between the developed and developing instead of stigmatization.

#### 4.2.2. Subjective Norm

We also found that when researchers enter the Roma community, they have to be aware of the powerful relations that can prevent community members from speaking and outsiders from listening [77]. There are also serious doubts, in the light of our findings showing the importance of group pressure, about the ameliorative and top-down approaches to health questions that are applied in vulnerable communities but which do not support empowerment and often do not respect the customs and the way of thinking of Roma people [79].

Outdated racist concepts are also still present in the interpretations of failure of compliance or adherence within Roma settlements. The adoption of reasoning that is in direct contradiction with compliance is an integral feature of Roma communities. This subjective norm results in the absence of the means and motivation needed to comply [25]. Otham et al. [80] found a correlation between knowledge basis and willingness to comply with rules. A lack of knowledge and facts serves as a booster of subjective norms and subsumed beliefs. It also supports a further negative approach to requirements resulting from health laws [81]. Subjective norms have also been shown to have a positive effect on knowledge-sharing behavior [82]. Together with trust, perceived risk and attitude, this significantly influences behavioral intention [83].

#### 4.2.3. Perceived Behavioral Control

We have pointed out that Roma tend to link their non-compliance with their natural incapacities. This tendency is supported by the group pressure within the SASG, and the adopted behavioral patterns do not leave much space for any change in behavior [25]. The negative attitudes and prejudices of the majority only decrease the perceived behavioral control among Roma, which is contrary to the desirable behavior [56]. The ongoing focus on disadvantage and victimization may have an effect on the group self-perception and a consequence on its health-related behavior [69]. Skills, abilities, environmental barriers and facilitators of ethnically-based SASGs need to be assessed in order to fully understand when a certain behavior is likely to occur [84]. Social workers and healthcare professionals are able to support changes in behavior by explaining the laws and remodeling the behavioral patterns adopted by Roma, but both groups need to be very culturally competent to do so [85]. 

### 4.3. Strengths and Limitations

Our aim was to explore the determinants of Roma behavior in the field of compliance with health laws. We consider the interconnection of two theories [28,47] and their application in the field of Roma compliance with health laws to be the strength of this manuscript. This approach allowed us to look at the issue of non-compliance from a new perspective. We considered the concept of compliance in the field of health through the lens of intention, which showed us the differences between individual, group and national rules. Regarding group rules, we also used Moore’s SAF concept [28]. Ajzen’s TPB can be used as a tool for predicting future behavior when used in studying minorities and health issues. We are not aware of any other studies carried out in the field of non-compliance that use the concept of semi-autonomous field combined with the theory of planned behavior in other ethnic minorities; we consider this to be one of the strengths of the study.

The modest number of studies dealing with the issue of Roma health in general is one of the limitations of our study. Furthermore, we can mention some limitations related to TPB, such as the assumption that behavior is the result of a linear decision-making process and ignoring the possibility that it can change over time or that it does not take into account environmental and economic factors. 

### 4.4. Implications

As we found, and several authors have stated [48,77], we still need more relevant and demand-oriented research in this area to be able to provide evidence-based recommendations for policy and practice. Anti-Roma prejudice, inclusion, participation, intervention effectiveness, evaluation and resources all need to be addressed. There is also a lack of research in the field of legal accountability and empowerment, which can also create pressure on local governments and health authorities [86]. We further found the importance of group rules. This supports the findings of Belak and Filakovska Bobakova [87] that community-level health-promotion intervention programs can be an effective tool with a real impact on Roma health but only if used properly. Belak et al. [49] also points out the need for specific training of public health practitioners and clinicians. All these issues have a close association to the choice of an effective approach in the process of implementing change in the area of healthcare. Research of the application of a participatory approach in Roma settlements would also be an interesting opportunity. 

Future research should also focus on raising awareness and explaining the rules contained in health laws to the Roma and on building positive attitudes among them. Evaluating pilot projects performed as part of qualitative research could help to further unravel the pathway leading to this result.

## 5. Conclusions

We found that although the objective of being healthy is the same for Roma and non-Roma, the intentions of Roma people living in settlements often differ. Their attitudes, subjective norms and perceptions of recommended behavior in general differ from those in the non-Roma majority part of society. Health laws are set to cope with the needs of non-Roma people, whereas for Roma, it is often very difficult to comply with state requirements. Roma health status is influenced by many factors, both internal and external. Some of them are under volitional control, while others, such as the living environment, can be changed only by across-the-board changes in policies addressing this marginalized population.

Our findings also show that group beliefs and community traditions are stronger and overrule individual and state behavioral influence. From our point of view, the solution to the frequently occurring non-compliance of Roma with national health laws lies in strengthening the dialogue with Roma communities, in taking greater account of their needs and attitudes and in eliminating racial prejudices among the majority population. A community-based participatory approach, together with improving the living conditions with the equal and non-discriminatory cooperation of the Roma themselves, is desirable.
